# Variability and population genetic structure in *Achyrocline flaccida* (Weinm.) DC., a species with high value in folk medicine in South America

**DOI:** 10.1371/journal.pone.0183533

**Published:** 2017-08-22

**Authors:** Juliana da Rosa, Gabriela Gomes Weber, Rafaela Cardoso, Felipe Górski, Paulo Roberto Da-Silva

**Affiliations:** Department of Biological Sciences, Plant Genetics and Molecular Biology Laboratory, Universidade Estadual do Centro-Oeste, UNICENTRO, Guarapuava, Paraná, Brazil; National Cheng Kung University, TAIWAN

## Abstract

Better knowledge of medicinal plant species and their conservation is an urgent need worldwide. Decision making for conservation strategies can be based on the knowledge of the variability and population genetic structure of the species and on the events that may influence these genetic parameters. *Achyrocline flaccida* (Weinm.) DC. is a native plant from the grassy fields of South America with high value in folk medicine. In spite of its importance, no genetic and conservation studies are available for the species. In this work, microsatellite and ISSR (inter-simple sequence repeat) markers were used to estimate the genetic variability and structure of seven populations of *A*. *flaccida* from southern Brazil. The microsatellite markers were inefficient in *A*. *flaccida* owing to a high number of null alleles. After the evaluation of 42 ISSR primers on one population, 10 were selected for further analysis of seven *A*. *flaccida* populations. The results of ISSR showed that the high number of exclusive absence of loci might contribute to the inter-population differentiation. Genetic variability of the species was high (Nei’s diversity of 0.23 and Shannon diversity of 0.37). AMOVA indicated higher genetic variability within (64.7%) than among (33.96%) populations, and the variability was unevenly distributed (F_*ST*_ 0.33). Gene flow among populations ranged from 1.68 to 5.2 migrants per generation, with an average of 1.39. The results of PCoA and Bayesian analyses corroborated and indicated that the populations are structured. The observed genetic variability and population structure of *A*. *flaccida* are discussed in the context of the vegetation formation history in southern Brazil, as well as the possible anthropogenic effects. Additionally, we discuss the implications of the results in the conservation of the species.

## Introduction

To understand the genetic variability of domesticated or wild plants and animals by 2020 is one of the goals of the United Nations [1]. The aim is to improve biodiversity by safeguarding ecosystems, species, and genetic variability. To achieve this goal, it is necessary to study the largest possible number of species from the different biomes of the world with an emphasis on the understudied biomes. In the Atlantic Forest biome, despite its importance in maintaining the planet’s biodiversity, very few species have been focused in such studies [2].

Among the different types of vegetation that exist in the Atlantic Forest, the ancient grassy fields, known as "Campos de Altitude", form a forest–grassland mosaic in the southern portion of this biome, and are one of the less studied and most threatened vegetation in Brazil [3]. Relative to the species from forests, species from grassy ecosystems are poorly studied and conserved [4,5]. Furthermore, it is predicted that the medicinal species of these regions and ecosystems are highly vulnerable. Two factors have led to this situation: the lack of study of these species and the increased worldwide demand for medicinal plants resulting in direct removal of these species from their natural habitat [6]. The vulnerability of these species increases because often, the most commonly used medicinal plant species in certain regions of the world are unknown to genetic conservation researchers and therefore, are not considered for conservation.

*Achyrocline flaccida* (Weinm) DC. (Asteraceae) is a medicinal plant species found in the original formation of grassy ecosystems, at forest edges, and in regions of vegetation regeneration in the southern portion of the Atlantic Forest. The species is herbaceous, popularly known as "Macela" and is the most common *Achyrocline* species in south and southeast Brazil. It is one of the most commonly used native plant species in folk medicine in South America [7]. The infructescence of the species is used in tea preparations or infusions, and in pillows and blankets [8].

The harvest of *A*. *flaccida* is closely linked with popular culture in Brazil. For example, every year on Good Friday, before sunrise, families go to the fields to harvest *A*. *flaccida* infructescence. It is believed that the plant is most effective in the treatment of diseases when the material is harvested in this manner. *Achyrocline flaccida* is mainly used in the treatment of digestive system disorders [9–12] and for relaxation when used inside of the pillows and blankets [8]. The plant contains several secondary compounds such as flavonoids, flavones, and coffee acids, as well as essential oils and pigments used in the cosmetic and food industry [7,13,14]. The species exhibits biological activities such as antioxidant and cytoprotective activities, inhibitory effects on tumors, and mitogenic activity in spleen and antiviral cells. Despite the ecological, medicinal, and cultural importance of the species, population genetic data for *A*. *flaccida* is not available in the literature, which makes it difficult to reach conclusions about its real conservation status.

Information on the distribution of genetic variability within and between natural *A*. *flaccida* populations can help in conservation and effective management of overexploited plant species [15]. DNA-based molecular markers are very efficient and most preferable in the analysis of the variability and genetic structure in plant species because the information is accessed directly from the genome of the target species, allowing safe analysis without interference of environmental factors [16]. Microsatellite molecular markers are the most suitable for these types of studies; however, these markers are not currently available for *A*. *flaccida*. The transferability of microsatellite markers between related species has been successfully achieved [17–19] and can be an alternative to *A*. *flaccida*. Additionally, when there is no previous knowledge of microsatellite regions for the genome of the species, ISSR (inter-simple sequence repeat) markers can also be used as an alternative [20–23]. The ISSR markers have the advantage of analyzing multiple loci in a single polymerase chain reaction (PCR); in addition, they are very informative, reliable, and produce a high level of polymorphism [24].

Furthermore, when data on genetic variability and population structure is aligned with the geological and paleoclimatic history of the habitat of a given species, the results can help define the patterns of distribution of a species throughout the historical and present timeline. For this understanding, the individual study of each species is necessary, because the extrapolation of data from one species to another can often lead to inaccurate conclusions owing to physical or biological barriers that influence one species but not the other. Mäder et al. [25] concluded that *Calibrachoa heterophylla* populations differed because they were influenced by local geomorphological events along the coast of the state of Rio Grande do Sul and northern Uruguay, which could not be identified if only long distance models of isolation and/or other species were used.

Aiming to contribute to the reduction of the scientific gap regarding the genetic data of *A*. *flaccida*, this work sought to answer the following questions: 1—The transferability of microsatellite markers is an efficient strategy for obtaining genetic data from *A*. *flaccida*?; 2—ISSR markers are efficient for obtaining population genetic data in *A*. *flaccida*?; 3—The populations of *A*. *flaccida* of the Atlantic Forest are structured? and 4—Can high utilization of this species in folk medicine to influence the patterns of genetic variability in the natural populations. The answers to these questions will help determine the real state of *A*. *flaccida* conservation in the grassy ecosystems of southern Brazil and, if necessary, contribute to the elaboration of conservation strategies and rational exploitation of the species. Additionally, the data obtained here may help us understand the patterns of maintenance and expansion of the plant populations of the grassy ecosystems in the Atlantic Forest of southern Brazil from a historical perspective.

## Materials and methods

### Plant material and DNA extraction

In this study, seven populations of *A*. *flaccida* were collected throughout their distribution in the grassy ecosystems of the Atlantic Forest biome in southern Brazil and were named according to the county of origin (Table 1). For all populations, vouchers were made and identified using the key for the genus *Achyrocline* (Less.) DC. in Brazil proposed by Deble [26]. The number of plants sampled from each population and the characterization of the sampling sites according to original [27] and actual ecosystem [28] are shown in Table 1. From each population, leaves of plants that were spaced at least 20 m from one another were collected. The collected leaves were stored in silica gel until DNA extraction. DNA extraction was performed following the protocol of Doyle and Doyle [29]. The concentration and quality of each DNA sample was checked on a 0.8% agarose gel using standard concentrations of the λ phage DNA (50, 100, and 200 ng).

**Table 1:** Data from the populations of *Achryrocline flaccida* (Weinm.) DC. sampled from grassy ecosystems of the Atlantic Forest in south Brazil. N: Number of individuals sampled.

Sampling site/Population name	State	Geographic coordinates	N	Original ecosystem	Actual ecosystem
Marialva	PR	23°24'35.25"S 51°47'42.75'O	20	ASF^1^	Agriculture
Guarapuava	PR	25°22'12.81"S 51°30'25.44"O	20	Mosaic^2^	Pastures
Porto Barreiro	PR	25°34'31.25"S 52°23'55.20"O	19	Mosaic^3^	Agriculture
Xanxerê	SC	26°51'35.77"S 52°21'50.46"O	16	Transition^3^	Pastures
Campos Novos	SC	27°20'10.58"S 51°21'08.86"O	18	Mosaic^2^	Pastures
Coxilha	RS	28°05'37.18"S 52°16'23.36"O	18	Mosaic^2^	Agriculture
Panambi	RS	28°21'11.94"S 53°28'30.99"O	20	Mosaic^4^	Agriculture

^1^ Atlantic Semi-deciduous Forest;

^2^ Araucaria Forest and grassy ecosystems;

^3^ Atlantic Semi-deciduous Forest and Araucaria Forest.;

^4^ Atlantic Semi-deciduous Forest with enclaves of Araucaria Forest.

### DNA amplification using microsatellite primers

To identify microsatellite primer pairs to be evaluated for transferability to *A*. *flaccida*, a literature review was first carried out to identify primer transferability studies among Asteraceae species. Of the primer pairs identified, 15 were selected based on the phylogenetic proximity of *A*. *flaccida* to the species for which the primers were initially developed (Table 2). Preference was given to primers pairs that amplified a higher number of alleles and showed transferability to other Asteraceae species as reported in previous studies (Table 2). All of these primers pairs were designed to amplify regions of genomic DNA.

**Table 2:** The sequences and annealing temperatures (AT, °C) of 15 nuclear microsatellite primer pairs selected for transferability evaluation in *Achryrocline flaccida* (Weinm.) DC.

Loci	Primers sequence (5’- 3’)	AT °C	Source
SS20E	CACACAGACACTCAAAGCTTCA	50°C	[30]
	ACCCGCCCTAAAAATAAAGA		
SS24F	AGCTTTTCTTCGCCATTTCCTTCC	59°C	[30]
	AATTTGGTTACTGGGTTTTCTTGA		
Eari4–5	ATGATGGTGGTGATGAGAAGTC	59°C	[31]
	TGGGTTTCAATGGATTCAAAG		
Eari4–6	GCGGTTTGTGTAGAAGTCC	57°C	[31]
	ATCTCACTGGTGAATTTCAGAG		
19	TTACCCGACTTGCTGAAAGG	55°C	[32]
	CCTTGCGTATTTGCACTCCT		
CO189	AGAGTAAGCACGAGACCG	60°C	[33]
	AGAACTTTACCTCCCACA		
CO227	GTTCGTCACCCTTTTCTC	62°C	[33]
	ATCTGCACTTCATCTTCTTC		
23_(TG)3+7st	AACCCTAAATCGTATGTGTCTAGTG	58.2°C	[34]
	CCTCCTTCCGAGCTATGTAGA		
Amb82	AAACAACTAGTGTGTGTTTCAGTGTG	60°C*	[35]
	GTCTTCGGCCGTAAAATGAC		
Sg_2	TCTAAACTGTAAGTCTTTGATGAAACC	65°C*	[36]
	GCCGTCAATCCTTACAATCC		
Sg_6	TTTACCTTTGAATTGCGGC	65°C*	[36]
	GTTTAGTACCAATCAACCATGGGC		
Sg_8	TCCCTCTTTATTCTTTCAACAAACC	65°C*	[36]
	GTTT AACACCAACATTGCAATCCC		
ER-HAJZC	GGATATCGGTTTGGCTTGA	63°C*	[37]
	GGAATCCCTTCTCTTTCTGA		
Lho35	AACCATCGCTGCACATTC	57°C	[38]
	GCAACACCACCACTGACG		
Hsalz-12	AAGCATCTATGAGGGGACAAA	53.9°C	[39]
	AAAAATATGCTGCTGGAAGTT		

(*) PCR Touchdown.

For the transferability evaluation, the 15 primer pairs were first evaluated by PCR in 10 *A*. *flaccida* individuals. The amplification was performed in a final volume of 10 μL containing 1X PCR buffer, 3 mM MgCl_2_, 0.2 mM dNTP, 0.8 mM each of forward and reverse primers, 0.4 U Taq DNA Polymerase), 20 ng DNA, and ultrapure water (to complete the volume).

For each primer pair, the annealing temperature described in the literature was initially used (Table 2). When non-specific amplification occurred at these initial temperatures, the annealing temperature was increased by 2°C until specific product amplification was obtained or 60°C was reached. When no amplification occurred, the temperature was decreased by 2°C until amplification was obtained or 45°C was reached. Among the 15 primer pairs, Amb82, Sg-2, Sg-6, Sg-8, and ER-HAJZC, did not require a change in the initial annealing temperature.

The amplified products were separated by electrophoresis in a 3% agarose gel at a constant 110 V current for 4 h and visualized under UV light by staining with ethidium bromide (0.5 μg/mL^-1^). To determine the size of the amplified fragments, a DNA ladder 100–bp marker was used.

The primers that showed amplification of fragments of expected sizes were considered positive amplification, but only those that presented a polymorphism were considered transferable to *A*. *flaccida*. However, the primers that presented non-specific amplification or did not amplify a product were considered inefficient for genetic studies in *A*. *flaccida*.

For the evaluation of the usefulness of transferred microsatellite primer pairs as genetic markers in *A*. *flaccida*, the primers were evaluated in seven populations. PCR and electrophoresis were performed as described previously.

### Amplification using ISSR markers

The evaluation of *A*. *flaccida* populations using ISSR markers was performed in two steps: Step 1: First, the best ISSR primers for genetic studies in *A*. *flaccida* were selected. The DNA of 20 genotypes of the population from Guarapuava, Brazil, was amplified using 42 ISSR primers from the UBC series (University of British Columbia) (Table 3). Step 2: Population genetic data were obtained from the seven populations of *A*. *flaccida*. After the analysis of the data obtained in Step 1, the best ISSR primers were identified (Table 3) and used to amplify DNA from 131 plants from the seven populations. In both steps, PCR amplification reactions were conducted in a final volume of 12.5 μL that contained 20 ng DNA, 0.2 μM each primer, 200 μM dNTPs, 1.5 mM MgCl_2_, 1 U Taq DNA Polymerase, 1X PCR buffer, and water to complete the volume. The thermocycler program consisted of an initial denaturation step at 94°C for 5 min, followed by 35 cycles of 90°C for 45 s, annealing temperature of the primer (Table 3) for 45 s, and 72°C for 60 s. At the end of the cycles, a final step of 72°C for 7 min was added for the complete extension of the fragments. The amplification products were separated by electrophoresis on 1.8% agarose gel at a constant voltage of 110 V for 2 h. The gels were stained with ethidium bromide and visualized under UV light. To determine the size of the amplified fragments, a 100-bp DNA ladder was used.

**Table 3:** The 42 ISSR primers and their respective attributes in *Achryrocline flaccida* (Weinm.) DC. AT: annealing temperature; AP: amplification product; NAF: number of amplified fragments, % P: percentage of polymorphism, PIC: polymorphic information content, MI: marker index, RP: resolving power.

ISSR*	AT	AP	NAF	%P	PIC	MI	RP
**UBC 807**	52	✓	12	83.33	0.37	5.71	5.40
**UBC 808**	50	✓	11	100.0	0.38	8.30	6.12
UBC 809	55	✓	6	66.67	0.41	6.81	2.40
UBC 810	52	✓	9	100.0	0.33	6.59	4.32
UBC 811	52	-	-	-	-	-	-
UBC 813	50	✓	10	60.00	0.33	4.58	2.50
UBC 814	50	✓	7	85.71	0.38	7.11	3.18
UBC 815	52	-	-	-	-	-	-
UBC 817	52	✓	13	76.92	0.29	4.25	3.40
UBC 820	52	✓	12	83.33	0.30	4.76	3.90
UBC 822	53	✓	5	80.00	0.35	5.81	2.00
UBC 823	52	✓	21	80.95	0.30	5.04	7.00
UBC 824	50	✓	7	42.86	0.31	2.74	1.22
UBC 826	52	✓	8	75.00	0.33	4.92	2.74
**UBC 827**	53	✓	14	78.57	0.37	6.50	6.59
UBC 828	50	✓	3	66.67	0.34	4.52	1.16
**UBC 834**	52	✓	16	93.75	0.41	8.04	9.78
**UBC 835**	52	✓	16	93.75	0.30	5.54	5.89
UBC 836	53	✓	13	84.62	0.30	5.05	4.53
UBC 840	50	-	-	-	-	-	-
UBC 843	54	✓	9	84.62	0.30	5.05	4.53
**UBC 848**	55	✓	18	72.22	0.39	5.49	7.68
UBC 852	50	-	-	-	-	-	-
**UBC 855**	55	✓	10	70.00	0.37	5.14	4.00
UBC 856	55	✓	6	83.33	0.32	5.03	2.60
**UBC 857**	54	✓	14	71.43	0.39	5.48	5.89
UBC 858	50	✓	11	54.55	0.37	4.50	3.29
UBC 859	55	✓	4	50.00	0.28	3.13	0.71
UBC 860	52	✓	4	75.00	0.32	4.95	1.33
UBC 861	52	-	-	-	-	-	-
UBC 864	55	-	-	-	-	-	-
**UBC 866**	55	✓	14	85.71	0.38	6.82	6.89
UBC 868	50	✓	5	60.00	0.49	6.53	2.71
UBC 873	50	✓	8	75.00	0.30	4.24	2.60
UBC 878	52	-	-	-	-	-	-
UBC 881	53	✓	9	100.0	0.34	7.07	4.33
UBC 886	55	✓	18	88.89	0.31	5.17	6.50
UBC 889	54	✓	11	72.73	0.35	5.58	3.89
**UBC 890**	54	✓	14	92.86	0.43	8.90	9.22
UBC 891	54	✓	17	88.24	0.43	9.41	3.33
UBC 899	50	✓	12	66.67	0.32	4.66	3.29
UBC 900	50	✓	8	12.50	0.23	0.72	0.27

^✓^positive amplification;—no amplification

* ISSR highlighted in bold were selected for genetic studies in *A*. *flaccida*.

### ISSR primers selection

To select the best ISSR primers for *A*. *flaccida* in Step 1, each amplified locus was evaluated according to the presence (1) or absence (0) of the DNA fragments (band). Based on the binary matrix obtained, the discriminatory power of ISSR primers was evaluated using three parameters: (i) polymorphism information content (PIC), (ii) marker index (MI), and (iii) resolving power (RP).

The PIC for each ISSR loci was computed as PIC*i* = 2ƒ_*i*_ (1 − *f*_*i*_); where PIC*i* is the polymorphic information content of the marker ‘*i*,’ ƒ_*i*_ is the frequency of the amplified locus (band present), and 1—ƒ_*i*_ is the frequency of the null allele [40].

MI was calculated as described in Varshney et al. [41]: MI = PIC x EMR, where EMR is “the effective multiplex ratio (*E*) and is defined as the product of the total number of loci per primer (*n*) and the fraction of polymorphic loci (β) (*EMR* = *n*. β).”

The RP was calculated according Prevost and Wilkinson [42]: RP = ∑*I*_b,_ where, *I*_b_ represents how informative the locus is of the polymorphism. The *I*_b_ can be translated into a 0–1 scale using the formula: *I*_b_ = 1 − (2 × |0.5 –p|), where, p is the proportion of the genotypes showing the locus.

The selection of the 10 best ISSR primers for genetic studies in *A*. *flaccida* was performed as follows: first, the 10 primers that showed the highest values for each attribute (PIC, MI, and RP) were identified separately. Then, 10 ISSR primers were identified according the following criteria in the following order: 1. primers that showed the highest values for PIC, MI, and RP, 2. primers that showed the highest values for PIC and PR, 3. primers that showed the highest values for MI and RP. Whenever a primer was selected, it was excluded before applying the next criteria.

### ISSR genetic population data

The 10 best ISSR primers identified for *A*. *flaccida* were evaluated by PCR in the seven populations. Each locus amplified in each population was evaluated according to the presence (1) or absence (0) of a product band.

For the analysis of the distribution pattern of each locus in the *A*. *flaccida* populations, the exclusive presence and exclusive absence of each locus in each population was determined. Then, the loci were grouped to construct a graphical distribution of the genetic variability with respect to population and sampling sites. For this, the loci were grouped according to the pattern of its presence or absence in the populations. After obtaining the loci groups for each population, the total of possible occurrences (possible bands) for all groups of loci and the number of occurrences observed in each group were computed. The number of occurrences observed for a group of loci was divided by the total possible occurrences in all groups of loci in a population to generate the frequency for that loci group within the population. The frequency values obtained for each group of loci were used to construct the graphs for the distribution and frequency of the loci groups in the populations.

The percentage of polymorphism (P), the Shannon diversity index (I), the Nei's genetic diversity (h), the Nei’s genetic distance (D), the coefficient of genetic differentiation (G_*st*_), and gene flow (Nm) among the populations were calculated using the POPGENE software version 1.32 [43]. A dendrogram was constructed based on the Nei’s genetic distance with UPGMA (unweighted pair group method using arithmetic averages).

Analysis of molecular variance (AMOVA) and the genetic differentiation index (F_*ST*_) were performed using the Arlequin software version 3.11 [44]. Mantel test was used to verify the correlation between of Nei’s genetic distance and geographical distance [45] with 1,000 permutations using the NTSYS 2.01 software. The principal coordinates analysis (PCoA) for the observation of the graphical distribution of the samples was performed according to the genetic correlation with the NTSYS 2.01 software.

To detect the genetic structure at the population level, Bayesian analysis was performed using Structure software [46]. In order to determine the ideal number of clusters (K), simulations were performed assuming that any number of clusters could be obtained between 1 and 10, with each simulation repeated 10 times. For this analysis, the no admixture ancestry model was used and the allelic frequencies were correlated by 10,000 burn-in and 100,000 MCMC (Markov chain Monte Carlo) repeats after burn-in. The Structure Harvester program [47] was used for the definition of K (number of clusters—genetic groups), which is more probable than those proposed by the analysis the criteria suggested by EVANO et al. [48].

## Results

### Transferability of microsatellite markers

Of the 15 microsatellite primer pairs evaluated, 10 (SS20E, SS24F, Eari4-5, Eari4-6, CO189, Sg-2, Sg-8, ER-HAJZC, LHO35, and HSALZ-12) amplified fragments of expected sizes after optimizing the annealing temperatures. However, when these 10 loci were evaluated in the *A*. *flaccida* populations, over 80% of the individuals showed null alleles for all the loci in all the populations, indicating that these loci were not suitable for obtaining the genetic data of *A*. *flaccida*.

### ISSR primers selection

Of the 42 ISSR primers evaluated in *A*. *flaccida*, 35 primers (83.3%) showed DNA amplification (Table 3). The total number of amplified fragments was 375 with a mean of 10.7 fragments per primer. The PIC, MI, and RP values for each primer are shown in Table 3. Based on the PIC, MI, and RP data, the primers identified as more informative for genetic studies in *A*. *flaccida* were UBC 890, UBC 834, UBC 866, UBC 808, UBC 827, UBC 807, UBC 857, UBC 848, UBC 855, and UBC 835 (Table 3).

### Genetic variability

The 10 ISSR primers identified in the first stage of this study amplified 221 loci when evaluated in the seven populations of *A*. *flaccida*. Of these, 190 (85.97%) were polymorphic in all populations. The degree of polymorphism did not show a high variation among populations, with the lowest and highest values of 55.2% and 63.8% in the Porto Barreiro and Campos Novos populations, respectively.

**Fig 1:**
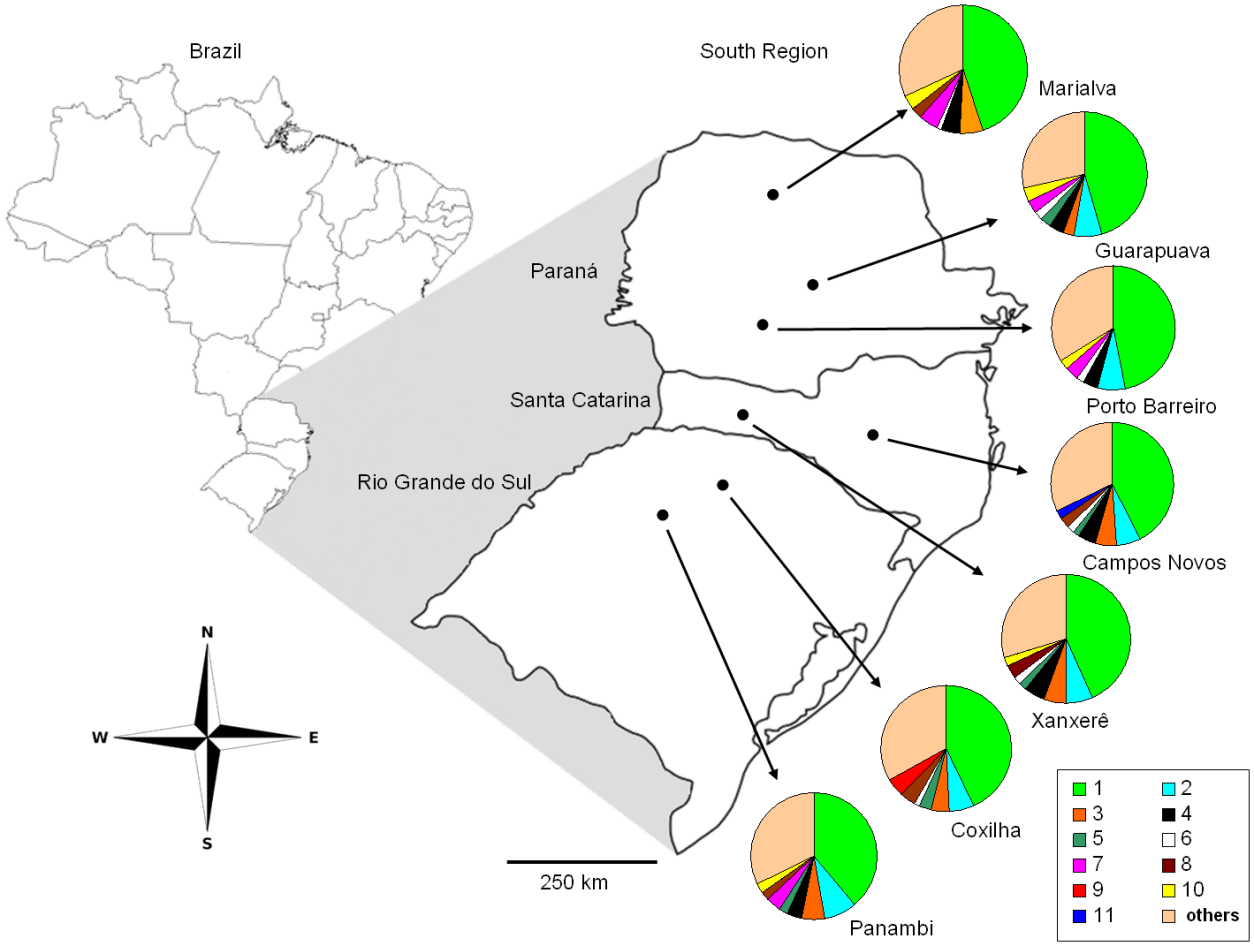
Distribution of ISSR loci group in *Achyrocline flaccida* (Weinm.) DC. populations from southern Brazil.

**Fig 2:**
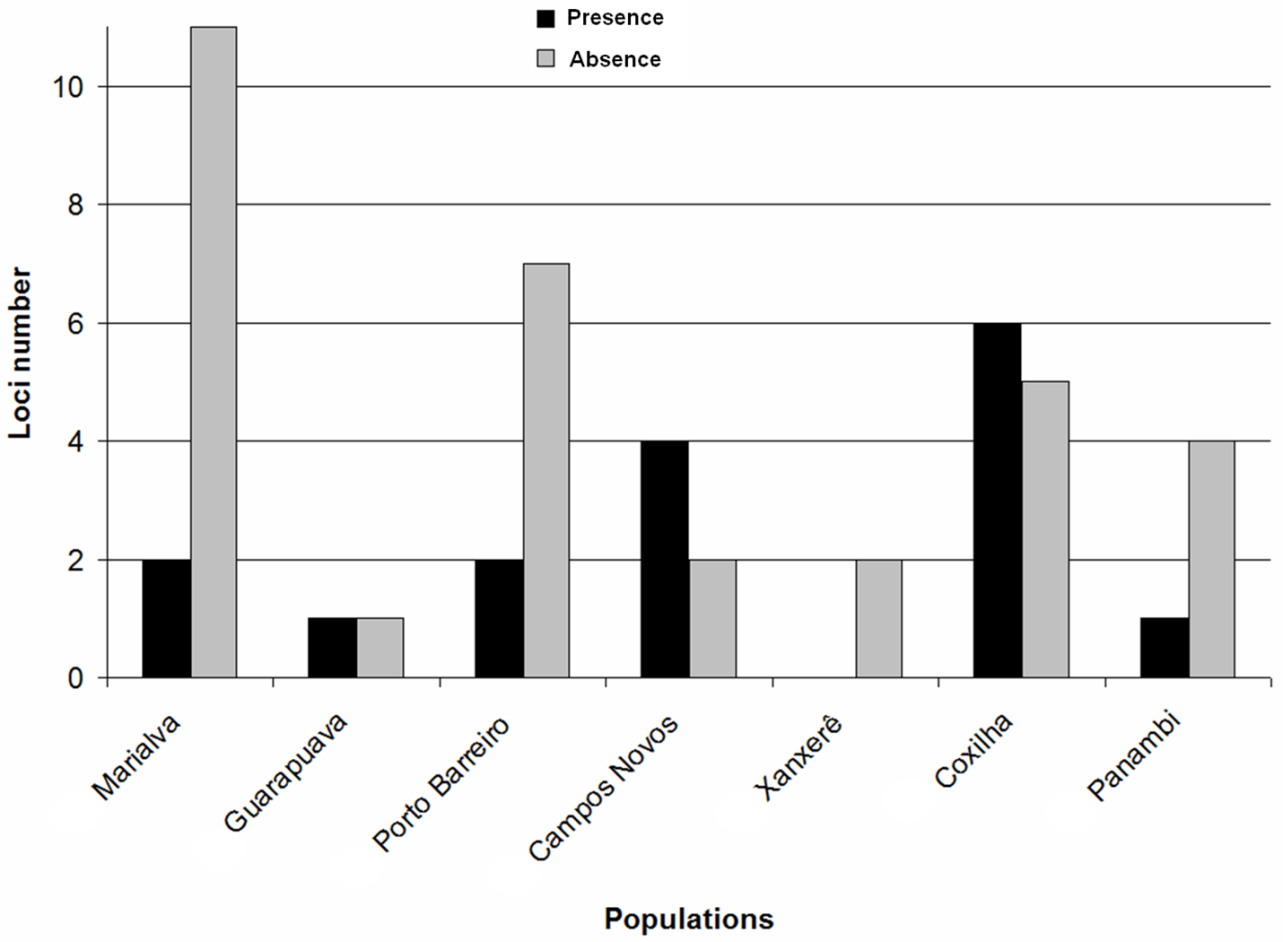
Exclusive presence and exclusive absence of ISSR loci in *Achyrocline flaccida* (Weinm.) DC. populations.

Of the 190 loci evaluated, 99 formed 11 groups with at least four loci per group. The largest group comprised 44 loci present in all populations and the smallest group comprised four loci. The remaining 91 loci had no similar pattern of distribution in the sampled populations and are referred to here as “others”. The analysis of the loci pattern allowed us to graphically visualize the distribution of genetic variability in the populations (Fig 1). The highest number of exclusive absence and exclusive presence of loci were observed in the of Marialva and Coxilha populations, respectively (Fig 2).

The Nei’s genetic variability of the populations ranged from 0.14 (Marialva) to 0.20 (Panambi), with all populations average of 0.23. The Shannon genetic variability index (I) ranged from 0.23 (Marialva) to 0.31 (Panambi) and all populations average of 0.37 (Table 4).

**Table 4:** Genetic variability in populations of *Achyrocline flaccida* (Weinm.) DC. sampled from grassy ecosystems in the Atlantic Forest of southern Brazil. h: Nei’s genetic variability; I: Shannon genetic variability index; P%: percentage of polymorphic loci.

Population	h	I	P%
Marialva	0.14	0.23	56.56
Guarapuava	0.18	0.28	62.44
Porto Barreiro	0.17	0.26	55.20
Xanxerê	0.16	0.25	57.01
Campos Novos	0.18	0.28	63.80
Coxilha	0.16	0.26	59.28
Panambi	0.20	0.31	63.35
All	0.23	0.37	100.0

### Population genetic structure

The AMOVA showed that the greatest variation was within the populations (66.71%) compared to the variation among populations (33.29%). G_*ST*_, the index of the genetic differentiation that considers how the genetic diversity is distributed among the populations was 0.33. The correlation between the Nei’s genetic distances and the geographical distances (in km) between populations (Table 5) was positive and significant (r = 0.77; p < 0.01), as estimated by the Mantel test.

**Table 5:** Nei's genetic distance (below diagonal) obtained with the ISSR marker data and geographic distance (in km) (above diagonal) between the populations of *Achyrocline flaccida* (Weinm.) DC. sampled from grassy ecosystems in the Atlantic Forest of southern Brazil.

Populations	GU	CN	XA	PB	PA	CO	MA
Guarapuava (GU)		211	186	81	380	312	210
Campos Novos (CN)	0.057		129	215	238	136	439
Xanxerê (XA)	0.050	0.073		153	190	140	380
Porto Barreiro (PB)	0.035	0.072	0.067		335	291	232
Panambi (PA)	0.085	0.089	0.110	0.105		115	558
Coxilha (CO)	0.106	0.081	0.107	0.120	0.136		520
Marialva (MA)	0.058	0.065	0.052	0.075	0.114	0.109	

Gene flow (Nm) among all *A*. *flaccida* populations was 1.39. In pairwise comparisons, the populations showed variable Nm values (Table 6). The greatest gene flow was observed between the Guarapuava and Porto Barreiro populations (5.20) and the least was between the Marialva and Coxilha populations (1.68). It was also observed that the higher the Nm, the lower the G_*st*_ values (Table 6).

**Table 6:** Gene flow Nm (below) and G_*st*_ (above) among *Achyrocline flaccida* (Weinm.) DC. populations sampled from grassy ecosystems in the Atlantic Forest of southern Brazil.

Populations	GU	CA	XA	PO	PA	CO	MA
Guarapuava (GU)		0.13	0.12	0.09	0.16	0.20	0.14
Campos Novos (CN)	3.50		0.16	0.15	0.16	0.17	0.15
Xanxerê (XA)	3.69	2.66		0.15	0.20	0.22	0.14
Porto Barreiro (PB)	5.20	2.79	2.80		0.19	0.23	0.17
Panambi (PA)	2.69	2.55	2.01	2.18		0.23	0.22
Coxilha (CO)	1.95	2.46	1.81	2.71	1.69		0.23
Marialva (MA)	3.08	2.78	3.18	2.42	1.85	1.68	

**Fig 3:**
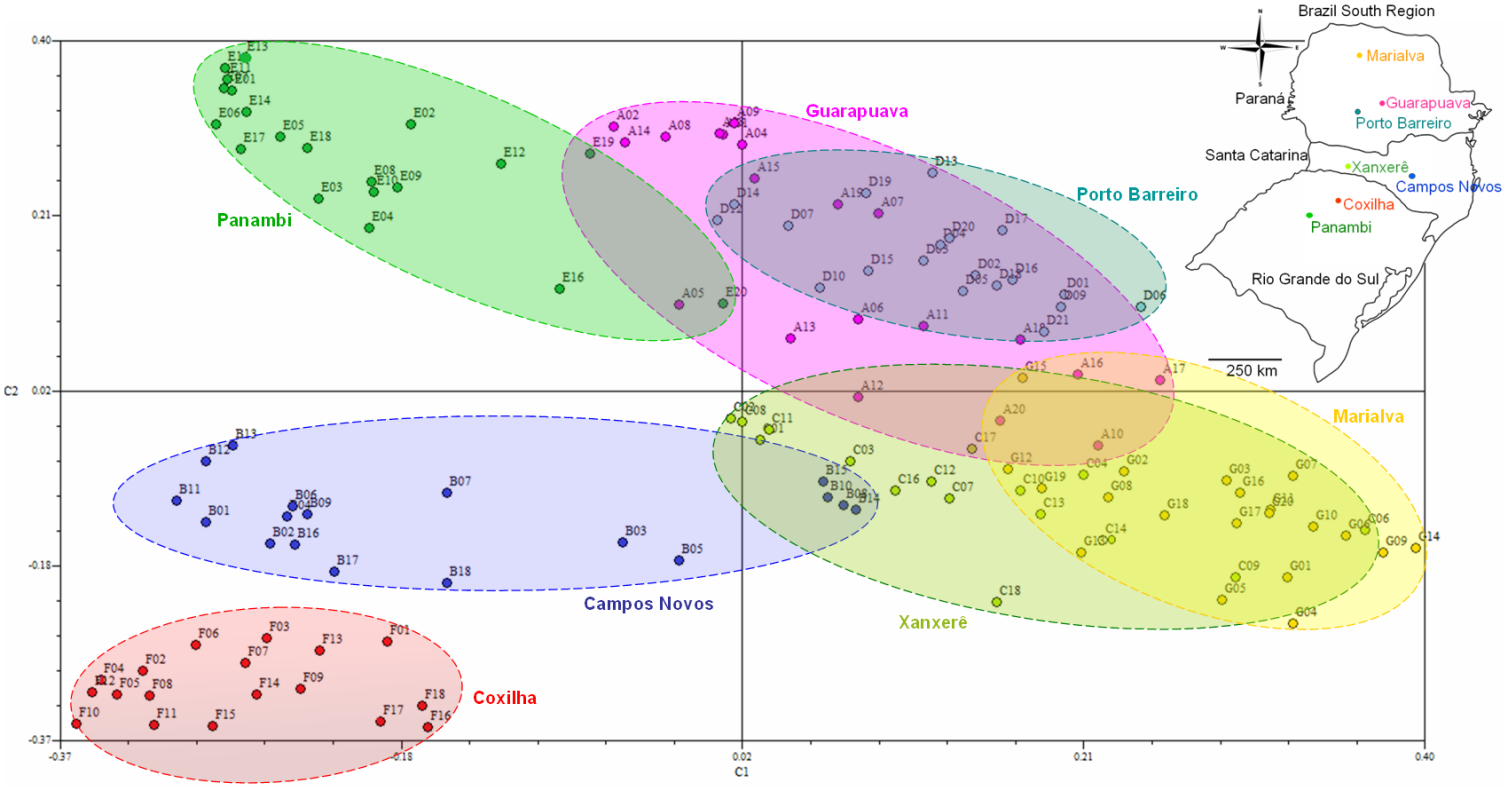
Principal Coordinate Analysis (PCoA) of *Achyrocline flaccida* (Weinm.) DC. populations from southern Brazil. The circles indicate the area occupied by each population in the quadrants.

The PCoA allowed for the visualization of the spatial structure of the population distribution. Thus, the populations Coxilha, Panambi, and Campos Novos were isolated in quadrants two and three, although Panambi and Campos Novos contained individuals that were far from the center of the population cluster (Fig 3). The Guarapuava and Porto Barreiro, and Xanxerê and Marialva populations overlapped by more than 85% of their occupied areas in the quadrants (Fig 3).

**Fig 4:**
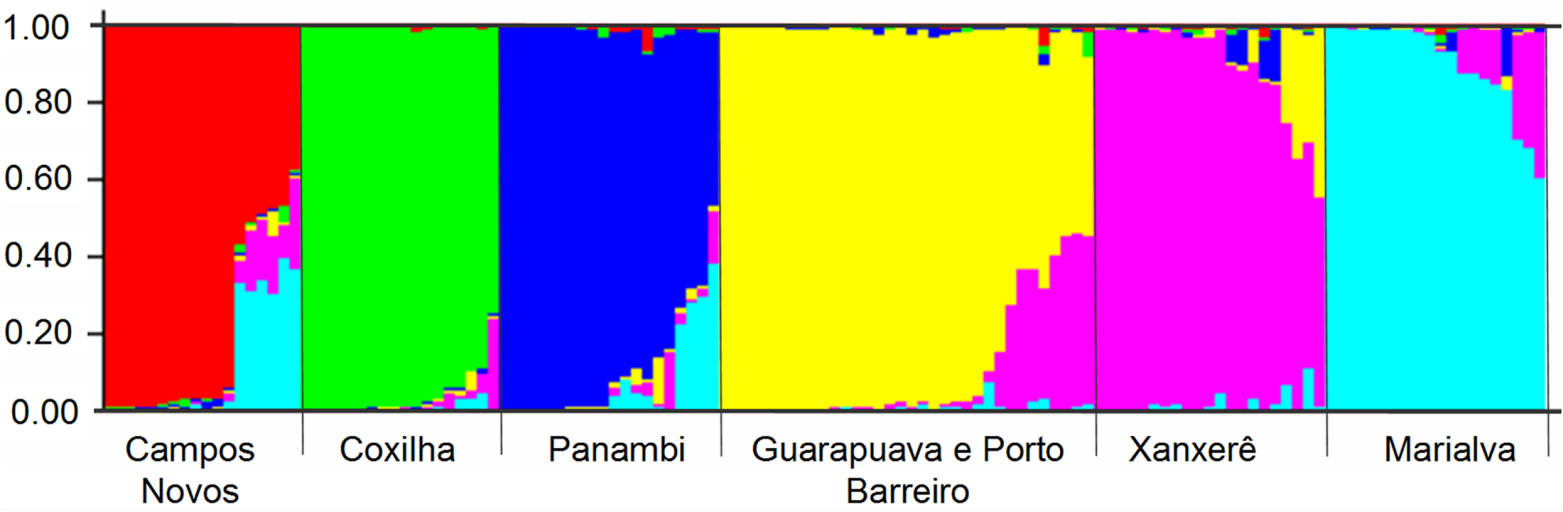
Genetic groups of *Achyrocline flaccida* (Weinm.) DC. established by Bayesian analysis using 190 ISSR loci.

In the Bayesian simulations of the Structure program, the number K (clusters, genetic groups) was defined as six. The distribution of individuals in the six genetic groups obtained (Fig 4) showed that the Porto Barreiro and Guarapuava populations belong to the same genetic group, whereas the other five populations are distinct genetic groups.

**Fig 5:**
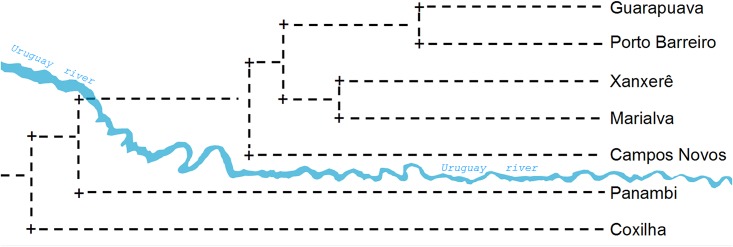
Dendrogram based on the Nei’s genetic distance of *Achyrocline flaccida* (Weinm.) DC. populations. The Uruguay River valley delimited two population groups.

In the dendrogram drawn from the Nei’s genetic distances [49], the Guarapuava and Porto Barreiro populations were the closest related, and along with the Marialva, Xanxerê, and Campos Novos populations, formed a delimited group (Fig 5).

## Discussion

### Transferability of microsatellite markers

In Myrtaceae and Rosaceae species, the transferability of microsatellite markers has been extensively explored and proven effective [19,50–53]. This approach has facilitated and accelerated the availability of molecular genetic data for species in absence of their own microsatellite markers. Therefore, it was predicted that the Asteraceae microsatellite primers could be useful in characterizing the *A*. *flaccida* populations; however, although Asteraceae primers were selected from the literature based on phylogenetic proximity, reports of transferability, and numbers of amplified alleles, they were not transferrable to *A*. *flaccida*. It is interesting that when these microsatellite primers were tested in 10 samples, 66.66% amplified fragments of expected size; however, when they were evaluated in whole populations, these primers were monomorphic and/or had a high number of null alleles. Notably, in the majority of studies that developed microsatellite primers, the authors tested these primers on a small number of samples from other species, which may falsely confirm their transferability. As with *A*. *flaccida*, these primers may have an excess of null alleles and/or be monomorphic when evaluated in populations with higher numbers of individuals and therefore, are not useful in population genetic studies. The results obtained in this study reinforce the need for the development of microsatellite primers specific to *A*. *flaccida*. As the development of microsatellites is expensive, and our group has been developing works with ISSR markers, we used these markers to get faster and more economical population genetic data of *A*. *flaccida*.

### ISSR primer selection

The choice of the best ISSR primers is an essential step in obtaining robust data for genetic studies and for this selection, the use of statistical indices should be prioritized by visual evaluation. In this study, the evaluation of PIC, MI, and RP data allowed the selection of the 10 most informative primers for genetic variability studies in *A*. *flaccida* (Table 3). These 10 primers amplified 190 polymorphic loci in the seven *A*. *flaccida* populations. This number is considered adequate because according to Colombo et al. [54], seven to 30 primers that amplify between 50 and 200 polymorphic fragments are sufficient for genetic diversity studies within a species.

### Genetic variability

The percentage of polymorphisms has been used as a measure of variability. In Asteraceae, variable values of polymorphism were found: 98.78% in *Aster spathulifolius* [55], 70.0% in *Carthamus tinctorious* [56], and 65.05% in *Nouelia insignis* [57]. Thus, the values of *A*. *flaccida* (85.97%) indicates high genetic variability in the species.

The frequency of ISSR loci in populations of *A*. *flaccida* was variable, with the populations of Marialva PR and Porto Barreiro PR indicating higher deviations in the number of loci and consequently, lower variability (Fig 1). This graphical representation of the distribution of genetic variability is presented for the first time in this study, and when we evaluated the numerical indices that represent the genetic variability and differentiation between the populations (Tables 4–6), we observed that the results were the same (high variability and differentiation between populations). This new form of presentation genetic variability results is an efficient and more visually friendly way to demonstrate genetic differentiation between populations.

The occurrence of exclusive loci in each population (Fig 2) had little variation and did not appear to contribute to the differentiation and distribution of variability among *A*. *flaccida* populations. The high exclusive absence of loci (11) in the Marialva population (Fig 2) seems to corroborate with data from the Shannon and Nei’s genetic variability analyses (low indices) of the population. Our previous observations in the last 25 years had not detected the presence of *A*. *flaccida* in the Marialva region therefore, it is possible that the species colonized this site in the last five years. The results for this population obtained in this study and absence of the species in the region for a long period, led to the hypothesis that this population is recent and suffered a possible founding effect.

### Genetic diversity and population structure

The parameters that quantify the genetic diversity, Nei’s genetic diversity (h), and Shannon diversity index (I) were detected at high levels in *A*. *flaccida* (h = 0.23; I = 0.37). These results are in agreement with the proposed by Hamrick and Godt [58], that generally geographically widespread species tend to maintain higher genetic diversity.

The distribution of the genetic diversity obtained by AMOVA showed higher variation within populations (66.71%) than among populations (33.29%), following the pattern observed in the literature for allogamous and/or pioneer species [59–61]. In addition, with AMOVA it was possible to observe that the genetic differentiation is high among populations (*F*_*ST*_ 0.33).

The comparison of gene flow and *G*_st_ (Table 6), and the correlation between the Nei’s genetic and geographical distances (r = 0.77, p < 0.01) among populations shows that *A*. *flaccida* populations follow the isolation-by-distance model. The estimated gene flow for a species may describe a migratory pattern of individuals occurring from the distant past to the present [57,62], and the data observed here should not be interpreted by considering only the current state of isolation in *A*. *flaccida* populations.

The Guarapuava and Porto Barreiro populations presented low genetic distance (Table 5), high gene flow (Table 6), they occupied the same area in the PCoA quadrants (Fig 3), and formed a single genetic group in the Bayesian analysis (Fig 4). This behavior leads to the hypothesis that these populations may assume a metapopulation model. The Marialva, Guarapuava, Porto Barreiro, Xanxerê, and Campos Novos populations were more similar in all analyzed variables, suggesting that they are more related than when compared to the Coxilha and Panambi populations. This finding is even more evident in the dendrogram (Fig 5). The populations of Xanxerê and Campos Novos are genetically closer and physically distant to Marialva population, while genetically distant and physically closer to Coxilha and Panambi. This observation indicates a possible geographic barrier to gene flow between the populations of Xanxerê/Campos Novos and Coxilha/Panambi.

The diversity and genetic structure of natural populations are affected by several factors including migratory capacity, mating systems, ecological characteristics of their habitats, altitude, and recent and historical events [15,63]. In *A*. *flaccida* the altitude was not considered because the populations of Xanxerê (777 m) and Marialva (700 m) are at similar altitude to Coxilha (750 m) and were still genetically distant. The analysis of the current ecosystems over the last 100 years and the history of these regions since the last glacial maximum led us to two complementary hypotheses for the diversity and genetic structure observed in *A*. *flaccida* populations.

**Fig 6:**
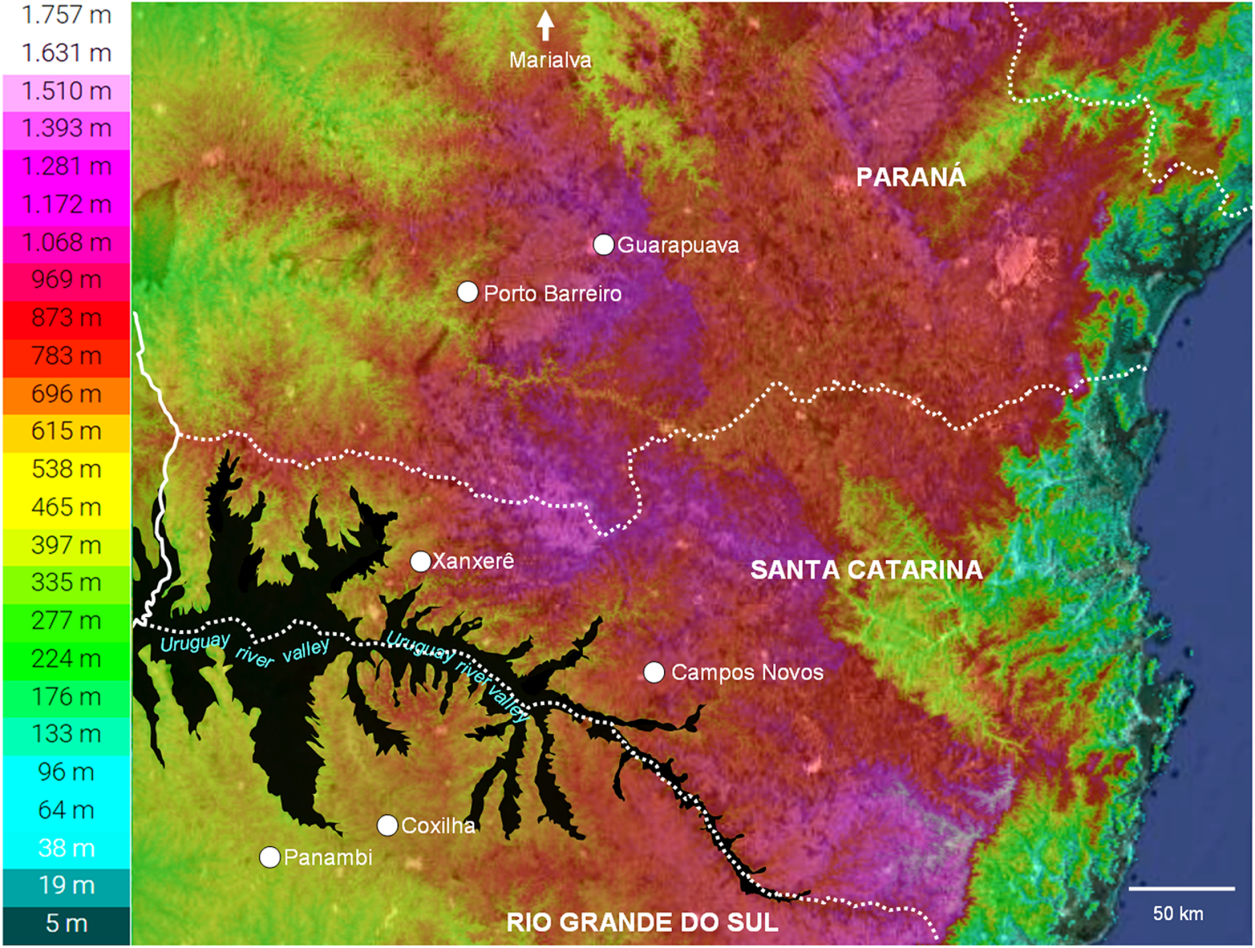
Topography of the southern region of Brazil. Highlight for the Uruguay River Valley (dark) and the sampling site of *Achryrocline flaccid* (Weinm.) DC. populations.

The first hypothesis is based on the historical dynamics of the vegetation in the southern fields of the Atlantic Forest and suggests an answer to why more closely related populations are more genetically distant. In order to understand the dynamics of the vegetation in the glacial and current periods, it is important to observe the topography of the regions where *A*. *flaccida* populations were collected. The Guarapuava, Xanxerê, and Campos Novos populations are in a high altitude continuum separated from the Coxilha and Panambi populations by the Uruguay River valley (Fig 6). This valley may have limited the gene flow between these populations.

The vegetation of the southern fields in the Atlantic Forest underwent several changes during the ice ages of the Lower and Middle Holocene period [64]. The decrease in temperature and humidity during these periods favored the expansion of grassy vegetation areas, which formed large continuous areas in the southern Brazilian states [64,65]. This condition favored the wide distribution of typical plant species of grassy vegetation areas and, according to Mayle et al. [66], among these species, the most abundant in these regions were those from the Poaceae and Asteraceae families; the latter being the family of *A*. *flaccida*. Behling [64] proposed that in the glacial periods during the Lower and Middle Holocene, the Araucaria Forest was isolated in refuges located in the river valleys. It is not difficult to conclude that these valleys may have also acted as a barrier to gene flow between populations of the species from grassy vegetation fields. Considering this context, it is possible that the genetic proximity observed between the *A*. *flaccida* from Guarapuava, Xanxerê, and Campos Novos populations is partly due to them having been somewhat continuous in the past. When analyzing the topography of the southern states of Brazil (Fig 6), it is possible to observe that such a situation was probable, because among these populations there are no river valleys that could have maintained forests acting as a barrier for the populations during cold and dry periods in the past. Additionally, the Uruguay River valley (Fig 6) may have acted as an important barrier for gene flow among the populations of Paraná/Santa Catarina and Rio Grande do Sul.

*Achyrocline*. *flaccida* populations from each region bounded by the Uruguay River valley experienced periods of physical continuity in the last maximum glacial period. The increase on temperature and humidity in this region during the upper Holocene between 5000 and 930 years ago, led to expansion of the Araucaria Forest [65,67,68] and isolation of populations due to grassy vegetation retraction. This may be one of the factors that contributed to the actual genetic differentiation observed in *A*. *flaccida* populations within each region. This hypothesis need to be tested further by adding data from other populations and using other species typical of the southern Atlantic Forest grassy vegetation field.

The second hypothesis that can also help to understand the pattern of genetic diversity and structure in the *A*. *flaccida* populations are human activities. In the last 100 years the isolation of the grassy vegetation has completely changed by deforestation and the establishment of pasture or agriculture, allowing the expansion of species such as *A*. *flaccida*. The populations of Porto Barreiro and Marialva are located in regions that were previously dense forests, and the diversity and structure data are consistent with the recent establishment of these populations in these areas.

Another factor that may influence the genetic patterns in A. *flaccida* is the dispersion of species by humans. The seed-bearing *A*. *flaccida* infructescence are widely used as medicine and in the manufacturing of pillows and blankets. There are companies that harvest and sell the infructescences throughout southern Brazil. The commercially available infructescences do not undergo processing that hinders germination. Seed germination tests of *Achyrocline satureioides*, a species in the same genus of *A*. *flaccida*, indicated that 25% of the seeds were still viable after 10 months of storage [69]. Then, the movement of seeds by marketing as well as long term seed viability could explain the establishment of a population where the species did not occur, as might be suggested for the Marialva and Porto Barreiro populations. Furthermore, this kind of seed flow may also increase gene flow which can contribute to the genetic similarity between distant populations, like Xanxerê and Marialva.

In conclusion, the results provided in this work show that: 1—the transferability of microsatellite markers is not an efficient strategy on *A*. *flaccida*; 2—the ISSR markers are a powerful tool to genetic studies on this species; 3—the populations of *A*. *flaccida* of grassy vegetation of southern Atlantic Forest have high genetic variability and are structured; 4—human activities (deforestation and folk medicine use) seems to influence the patterns of genetic variability in the natural populations of the species. The lack of previous genetic data of *A*. *flaccida* does not allow drawing any inferences about genetic drift on the species, but our results give support to the conclusion that the species is not at risk of extinction in the short term in the studied regions.
